# Early administration of magnesium sulfate and its impact on clinical outcomes in ICU-admitted patients with COPD: a retrospective cohort study

**DOI:** 10.3389/fphar.2025.1616294

**Published:** 2025-09-04

**Authors:** Min Xiao, Min Chen, Xuefeng Ding, Shan Lin

**Affiliations:** ^1^ Department of Critical Care Medicine, Affiliated Hospital of North Sichuan Medical College, Nanchong, Sichuan, China; ^2^ Department of Respiratory and Critical Care Medicine, Affiliated Hospital of North Sichuan Medical College, Nanchong, Sichuan, China

**Keywords:** magnesium sulfate, COPD, critical care, prognosis, MIMIC-IV

## Abstract

**Background:**

Magnesium sulfate is commonly utilized in critical care due to its vasodilatory, bronchodilatory, and neuroprotective properties. However, its impact on mortality outcomes in patients with chronic obstructive pulmonary disease (COPD) requiring intensive care remains inadequately defined.

**Methods:**

A retrospective cohort study was conducted on patients with COPD who were admitted to the ICU at Beth Israel Deaconess Medical Center in Boston from 2008 to 2019. Early administration of magnesium sulfate was considered for intravenous administration within 48 h of ICU admission. Propensity-score-based methods, such as inverse probability weighting, were employed to evaluate the correlation between early use of magnesium sulfate and 28-day mortality.

**Results:**

A total of 3,651 ICU admissions for COPD were included, of which 1,148 (31.4%) patients received magnesium sulfate within the first 48 h. Administering magnesium sulfate early was linked to a reduced 28-day mortality rate (hazard ratio 0.76, 95% confidence interval 0.60–0.95), with consistent results across predefined subgroups. This correlation remained consistent regardless of baseline serum magnesium levels and did not increase the risk of acute kidney injury (AKI). The calculated E-value of 1.96 indicates that significant unmeasured confounding factors would be necessary to fully account for the observed relationship.

**Conclusion:**

In this single-center retrospective cohort, early magnesium sulfate administration in critically ill patients with COPD was associated with lower 28-day mortality without an observed increase in AKI risk. These results advocate for prospective multicenter studies to validate these connections, investigate optimal dosing approaches, and pinpoint the patient subgroups most likely to benefit from this intervention.

## Introduction

Chronic obstructive pulmonary disease (COPD) stands as the primary cause of respiratory-related disability and mortality globally. Acute exacerbations often necessitate intensive care unit (ICU) admission for advanced life support, such as mechanical ventilation, due to severe complications like respiratory failure and infection (GBD Chronic Respiratory Disease Collaborators, 2017; GBD 2015 Disease and Injury Incidence and Prevalence Collaborators, 2016; Funk et al., 2013). Despite therapeutic progress, ICU patients with COPD exhibit significantly higher mortality rates compared to those without COPD. The prognosis is further compromised by issues like prolonged mechanical ventilation and ventilator-associated pneumonia (VAP) (Nseir et al., 2005). Remarkably, the occurrence of VAP can elevate ICU mortality rates in COPD patients to as high as 64%, while significantly prolonging the duration of mechanical ventilation and hospitalization. Although optimized ventilation strategies, including early invasive–non-invasive sequential therapy, have enhanced clinical outcomes, effective pharmacological interventions targeting mortality reduction remain a pressing area of investigation (Lei et al., 2023; Gungor et al., 2018).

Magnesium sulfate, a compound with bronchodilatory, anti-inflammatory, and electrolyte-modulating properties, has the potential to enhance outcomes in critically ill patients with COPD through various mechanisms (de Baaij et al., 2015). Patients in the ICU with COPD often exhibit hypophosphatemia, electrolyte imbalances, and systemic inflammatory responses that could be mitigated by the therapeutic effects of magnesium sulfate (Wozniak et al., 2022; Panahi et al., 2017). Additionally, noninvasive ventilation (NIV) has been proven to significantly decrease the occurrence of VAP. As a supplementary treatment, magnesium sulfate might indirectly reduce the VAP risk by diminishing the necessity for tracheal intubation (Hess, 2013; Lorente et al., 2007). In cases of respiratory failure, an imbalance between the energy supply and demand of the respiratory muscles contributes to respiratory fatigue. The bronchodilatory properties of magnesium sulfate could potentially alleviate this by lowering airway resistance and decreasing muscle workload (Roussos and Koutsoukou, 2003; Touyz et al., 2024). Previous studies examining the use of Mg in COPD exacerbations have yielded inconsistent results. A systematic review indicated that intravenous magnesium was linked to decreased hospital admission rates, shorter stays, and improved dyspnea scores during acute exacerbations (Jahangir et al., 2022; Liu et al., 2024; Ni et al., 2022). Conversely, other studies have not shown significant enhancements in crucial clinical outcomes. A non-Cochrane review concluded that while magnesium may boost the bronchodilatory effects of beta_2_-agonists, it does not notably impact dyspnea scores, hospital admission rates, or relapse rates (Shivanthan and Rajapakse, 2014). Given these inconclusive findings, current clinical guidelines do not advocate for the routine use of magnesium sulfate in acute COPD exacerbations.

This uncertainty, particularly in studies involving general ward patients or those with moderate disease, supports our hypothesis that any potential benefit of magnesium sulfate may be context-dependent. Critically ill patients with COPD in the ICU represent a population with more profound physiological derangements and a higher burden of complications, where standard treatments may be insufficient. By specifically evaluating the impact of magnesium sulfate in this high-risk subgroup, our study sought to clarify whether it can confer clinically meaningful benefits, such as reduced duration of ventilation, fewer complications, and lower mortality, which may have been obscured in broader patient populations. Addressing this knowledge gap could help reconcile conflicting evidence and determine whether ICU patients with COPD derive unique benefits from Mg therapy.

## Methods

### Data sources

This study utilized data from the Medical Information Mart for Intensive Care IV (MIMIC-IV), a comprehensive single-center database containing electronic health records of patients admitted to the Beth Israel Deaconess Medical Center in Boston, Massachusetts, United States, from 2008 to 2019 (Johnson A. E. W. et al., 2023; Johnson et al., 2024). Access to the database was approved by the Institutional Review Boards of the Beth Israel Deaconess Medical Center and the Massachusetts Institute of Technology in Cambridge, Massachusetts, United States (record ID: 49780033). Owing to data anonymization, patient consent was not required to adhere to ethical standards and privacy regulations.

### Study population

Participants were COPD patients aged 18 years and older who were admitted to the ICU under specific criteria: 1) a confirmed diagnosis of COPD, as indicated by a post-bronchodilator FEV1/FVC ratio of less than 0.70; 2) their first ICU admission; and 3) a minimum ICU stay of 48 h. Data collection included a wide range of parameters such as demographic details (age and sex), disease severity markers (Oxford Acute Severity of Illness Score (OASIS)), comorbidity burden (Charlson Comorbidity Index), organ support interventions (MV and continuous renal replacement therapy (CRRT)), baseline magnesium levels, and infection-related parameters (sepsis status and laboratory biomarkers). Laboratory indicators were initially measured within 24 h of ICU admission. For patients with multiple ICU admissions, only data from the first admission were considered to maintain data independence.

### Outcomes

The main endpoint was the effect of magnesium sulfate treatment within 48 h of ICU admission on 28-day mortality. Secondary endpoints comprised the incidence of acute kidney injury (AKI), ICU mortality rate, ICU stay duration, and MV duration.

### Covariate filtering methods

**FIGURE 1:**
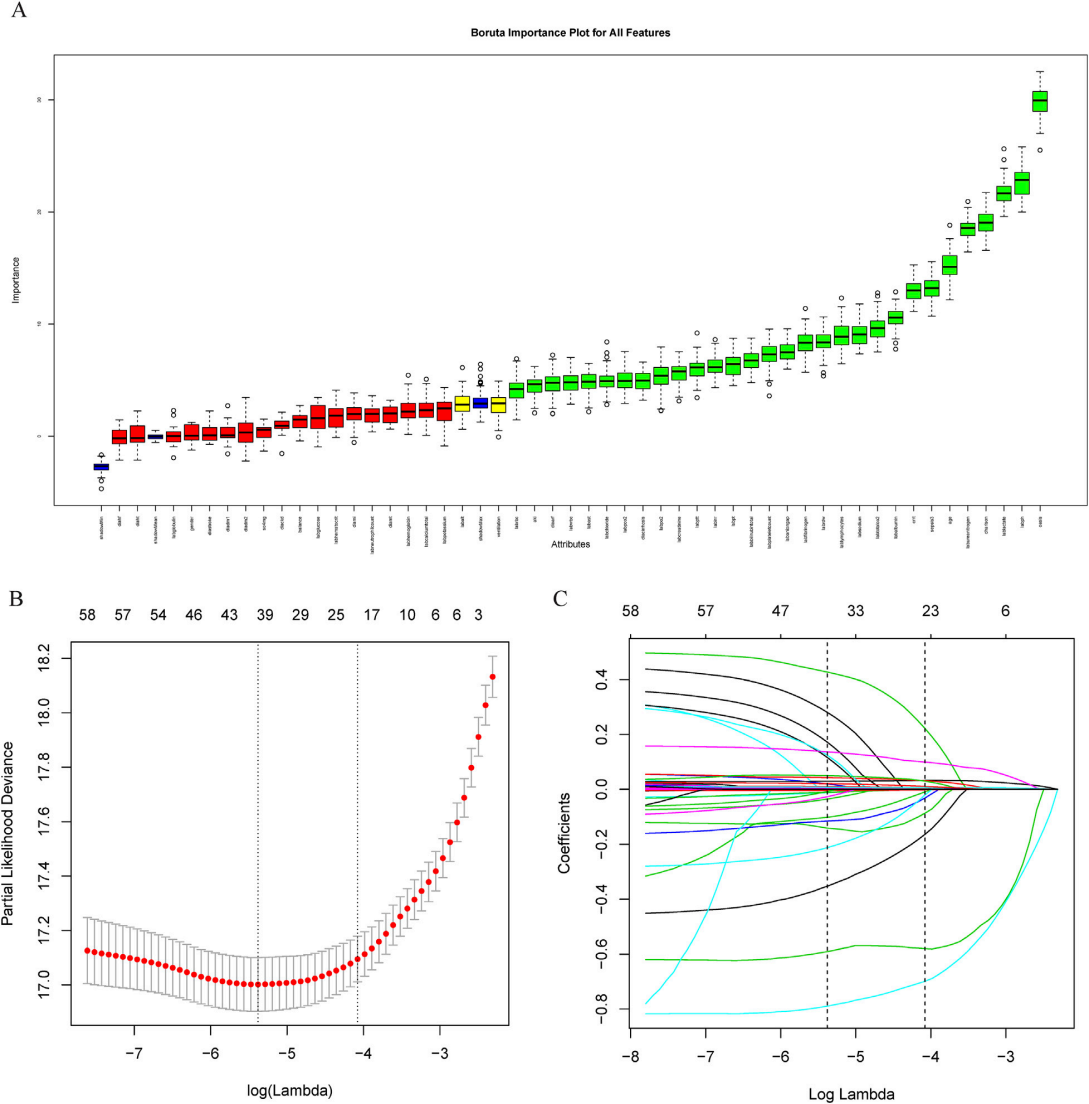
Feature variable screening. **(A)** Feature variable screening using Boruta; **(B, C)** Feature variable screening using LASSO. Abbreviations: LASSO, least absolute shrinkage and selection operator.

The covariate selection utilized the Boruta algorithm, least absolute shrinkage, and selection operator (LASSO) regression. LASSO regression employs L1 regularization to reduce certain feature coefficients to zero, aiding in both feature selection and parameter estimation. The Boruta algorithm functions as a robust wrapper method that statistically assesses original features against manually generated “shadow features” (randomized arrangements of the original data) using a Z-score test (Lin et al., 2025), effectively minimizing the false-positive rate. Ultimately, the variables “age, OASIS score, AKI, sepsis, MV, CRRT, blood urea nitrogen (BUN), creatinine” were incorporated into the regression model (Figure 1).

### Statistical analysis

Continuous variables are reported as mean ± standard deviation or median interquartile range, depending on their distribution. Groups of variables were compared using Student's t-test for normally distributed data and the Wilcoxon rank-sum test for non-normally distributed data. Categorical variables are presented as counts and percentages and were analyzed using the chi-square test.

Three models were constructed to evaluate the impact of early magnesium sulfate administration on 28-day mortality: Model 1 remained unadjusted, Model 2 was adjusted for characteristic variables, and Model 3 included a propensity score methodology involving matching, covariate-adjusted scores, and inverse probability weighting. E-values were computed to assess the potential impact of unmeasured confounders on the relationship between early magnesium sulfate use and 28-day mortality (VanderWeele and Ding, 2017; Haneuse et al., 2019). Survival disparities were examined utilizing the Kaplan–Meier method and evaluated through time-series tests. Stratified analyses and interaction tests were carried out to confirm the consistency of associations across various subgroups, encompassing demographics, therapeutic interventions, sepsis, AKI, baseline magnesium levels, and OASIS scores. Data analysis was conducted using R software (version 4.4.2), with p-values below 0.05 considered statistically significant.

## Results

### Basic characteristics of the study population

In this study, we analyzed a cohort of 3,651 patients with severe COPD. Within the first 48 h of ICU admission, 31.44% of the patients (1,148) received magnesium sulfate treatment, as detailed in Table 1. Our findings indicated that sepsis was diagnosed in 47.52% of the cohort (1,735 patients), and 71.87% (2,624 patients) developed AKI after admission. Both comorbidities occurred less frequently in the magnesium sulfate group than in the untreated group. This group also had significantly lower CRRT and invasive mechanical ventilation rates, as shown in Table 1.

**TABLE 1:** Characteristics of critically ill patients with COPD.

Variables	All patients (N = 3,651)	Without magnesium sulfate (N = 2,503)	With magnesium sulfate (N = 1,148)	P-value
Age	71.74 ± 10.92	71.69 ± 10.79	71.83 ± 11.20	0.727
Sex				0.579
Male	1963 (53.77%)	1,338 (53.46%)	625 (54.44%)	
Female	1,688 (46.23%)	1,165 (46.54%)	523 (45.56%)	
OASIS	31.28 ± 8.18	31.81 ± 8.34	30.13 ± 7.71	<0.001
Charlson score	6.75 ± 2.73	6.86 ± 2.74	6.50 ± 2.67	<0.001
MV	1,209 (33.11%)	916 (36.60%)	293 (25.52%)	<0.001
CRRT	155 (4.25%)	135 (5.39%)	20 (1.74%)	<0.001
AKI	2,624 (71.87%)	1926 (76.95%)	698 (60.80%)	<0.001
Sepsis	1735 (47.52%)	1,293 (51.66%)	442 (38.50%)	<0.001
Length of ICU stay (days)	4.24 (2.86–7.36)	4.21 (2.85–7.24)	4.53 (2.95–7.87)	0.237
Length of CRRT (days)	5.00 (3.00–9.00)	5.00 (3.00–8.00)	7.50 (3.75–13.50)	0.065
Length of MV (hours)	28.00 (11.58–82.57)	35.00 (13.96–90.83)	15.00 (7.13–45.30)	<0.001
ICU mortality	241 (6.60%)	202 (8.07%)	39 (3.40%)	<0.001
28-day mortality	546 (14.95%)	438 (17.50%)	108 (9.41%)	<0.001
Baseline blood Mg level (mg/dL)	2.09 ± 0.42	2.10 ± 0.43	2.09 ± 0.38	0.518
White blood cells (K/uL)	11.40 (8.30–14.90)	11.50 (8.40–15.10)	11.15 (8.07–14.48)	0.061
Red blood cells (m/uL)	3.55 (3.06–4.04)	3.53 (3.02–4.03)	3.57 (3.12–4.05)	0.027
Neutrophil (K/uL)	10.95 (10.90–10.95)	10.95 (10.95–10.95)	10.95 (10.76–10.95)	0.458
Lymphocyte (K/uL)	1.74 (1.38–1.74)	1.74 (1.36–1.74)	1.74 (1.46–1.74)	0.246
Platelet (K/uL)	191.00 (142.50–247.00)	193.00 (143.38–250.12)	187.75 (141.00–240.00)	0.111
Hematocrit (%)	32.70 (28.33–37.03)	32.60 (27.93–36.90)	33.00 (29.10–37.28)	0.011
Hemoglobin (g/dL)	10.50 (9.00–11.99)	10.40 (8.90–11.87)	10.60 (9.30–12.11)	<0.001
RDW (%)	14.70 (13.60–16.20)	14.80 (13.70–16.38)	14.56 (13.50–15.90)	<0.001
Albumin (g/dL)	3.14 ± 0.34	3.13 ± 0.35	3.16 ± 0.32	0.004
Globulin (g/dL)	2.94 ± 0.12	2.94 ± 0.13	2.94 ± 0.09	0.791
Sodium (mEq/L)	138.07 ± 4.81	138.13 ± 4.89	137.94 ± 4.65	0.263
Potassium (mEq/L)	4.32 ± 0.59	4.34 ± 0.58	4.28 ± 0.61	0.002
Calcium (mg/dL)	8.48 ± 0.67	8.49 ± 0.68	8.45 ± 0.66	0.124
Chloride (mEq/L)	101.93 ± 5.96	101.78 ± 6.09	102.26 ± 5.66	0.024
Glucose (mg/dL)	143.16 ± 56.27	143.69 ± 56.78	142.02 ± 55.15	0.407
pH	7.35 ± 0.05	7.35 ± 0.06	7.36 ± 0.05	0.018
PaCO_2_	46.87 ± 9.64	47.17 ± 10.12	46.22 ± 8.48	0.005
PaO_2_	109.67 ± 62.08	107.23 ± 59.64	114.98 ± 66.81	<0.001
Lactate (mmol/L)	2.02 ± 0.97	2.03 ± 0.99	2.01 ± 0.94	0.720
Prothrombin time (s)	15.31 ± 6.51	15.51 ± 6.83	14.85 ± 5.72	0.004
Fibrinogen (mg/dL)	305.99 ± 80.11	307.82 ± 82.80	301.99 ± 73.78	0.041
Partial thromboplastin time (s)	38.31 ± 18.84	38.61 ± 19.31	37.66 ± 17.77	0.161
INR	1.41 ± 0.58	1.42 ± 0.61	1.37 ± 0.52	0.005
Total bilirubin (mg/dL)	1.28 (0.55–1.28)	1.28 (0.50–1.28)	1.28 (0.60–1.28)	0.226
ALT (IU/L)	131.56 (21.00–131.56)	131.56 (20.00–131.56)	131.56 (23.00–131.56)	0.966
AST (IU/L)	213.53 (30.00–213.53)	213.53 (29.00–213.53)	213.53 (33.50–213.53)	0.601
BUN (mg/dL)	20.50 (14.00–31.59)	21.50 (14.50–34.00)	18.50 (13.00–28.00)	<0.001
Creatinine (mg/dL)	1.00 (0.73–1.44)	1.00 (0.75–1.52)	0.94 (0.70–1.30)	<0.001
Comorbidities				
Hypertension	1,387 (37.99%)	929 (37.12%)	458 (39.90%)	0.108
Type II diabetes	1,215 (33.28%)	844 (33.72%)	371 (32.32%)	0.404
Type I diabetes	35 (0.96%)	26 (1.04%)	9 (0.78%)	0.463
Heart failure	1,534 (42.02%)	1,076 (42.99%)	458 (39.90%)	0.079
Myocardial infarction	550 (15.06%)	396 (15.82%)	154 (13.41%)	0.059
Malignant tumor	696 (19.06%)	473 (18.90%)	223 (19.43%)	0.706
CKD	888 (24.32%)	635 (25.37%)	253 (22.04%)	0.029
Cirrhosis	214 (5.86%)	159 (6.35%)	55 (4.79%)	0.062
Stroke	324 (8.87%)	231 (9.23%)	93 (8.10%)	0.266

Abbreviations: OASIS, oxford acute severity of illness score; AKI, acute kidney injury; MV, mechanical ventilation; CRRT, continuous renal replacement therapy; CKD, chronic kidney disease; ICU, intensive care unit; ALT, alanine aminotransferase; AST, aspartate aminotransferase; BUN, blood urea nitrogen; COPD, chronic obstructive pulmonary disease.

### Clinical outcomes in critically ill patients with COPD

**FIGURE 2:**
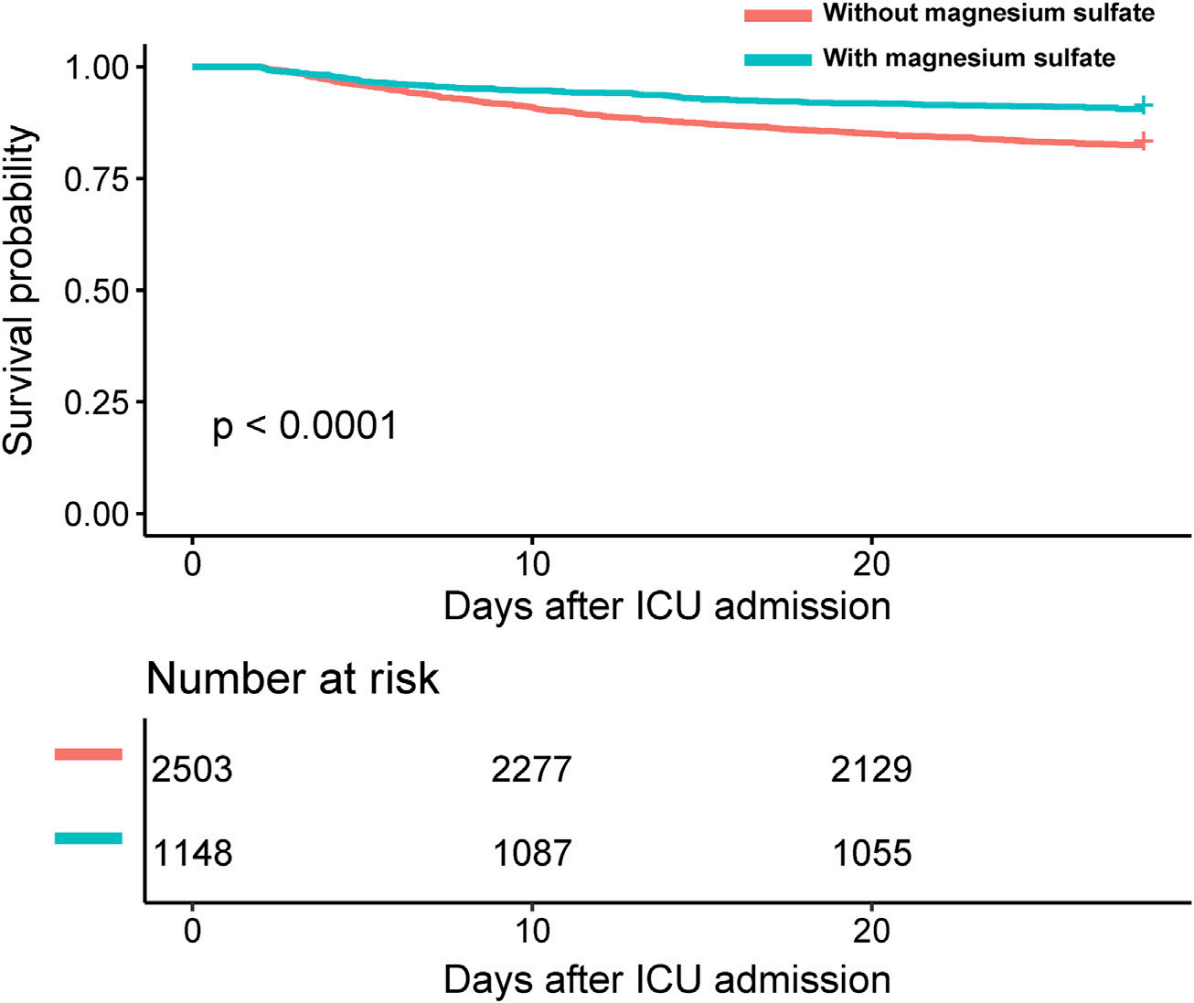
Kaplan-Meier survival curve. Abbreviations: ICU: intensive care unit.

Clinically, patients treated with magnesium sulfate demonstrated significantly lower mortality rates both in the ICU and at 28 days post-admission compared to those who did not receive the treatment, with p-values less than 0.05 for both endpoints (Table 2). Kaplan–Meier survival curves further emphasized a significantly higher 28-day survival rate among patients treated with magnesium sulfate, confirmed by a time-series test with a p-value of less than 0.05 (Figure 2). Additionally, the mean duration of MV was significantly shorter in the magnesium-treated group (p < 0.001). However, no significant difference was observed in the length of ICU stay between the two groups (p = 0.237).

**TABLE 2:** Clinical outcomes in critically ill patients with COPD.

Clinical outcomes	Without magnesium sulfate (N = 2,503)	With magnesium sulfate (N = 1,148)	*P*-value
ICU mortality, n (%)	202 (8.07%)	39 (3.40%)	<0.001
28-day mortality, n (%)	438 (17.50%)	108 (9.41%)	<0.001
Length of ICU stay (days)	4.21 (2.85–7.24)	4.53 (2.95–7.87)	0.237
Duration of MV (hours)	35.00 (13.96–90.83)	15.00 (7.13–45.30)	<0.001

Abbreviations: COPD, chronic obstructive pulmonary disease; ICU, intensive care unit; MV, mechanical ventilation.

### Impact of magnesium sulfate use on clinical outcomes

Table 3 demonstrates a consistent link between lower 28-day mortality rates in all examined models for patients receiving magnesium sulfate treatment. Particularly, in the model utilizing propensity score inverse probability weighting, the use of magnesium sulfate was linked to a 24% reduction in 28-day mortality compared to non-users (hazard ratio [HR] 0.76 [95% confidence interval {CI} 0.60–0.95]). The calculated E-value of 1.96 (upper confidence limit 2.89) indicates that an unmeasured confounding factor would need to have a relative risk of at least 1.96 to negate the observed association.

**TABLE 3:** Associations between magnesium sulfate use and clinical outcomes in the crude analysis, multivariable Cox analysis, and propensity score analyses.

Clinical outcomes			
28-day mortality	Groups	HR (95% CI)	*P*-value
Crude analysis	Without magnesium sulfate With magnesium sulfate	Ref. 0.52 (0.42–0.64)	- <0.0001
Multivariable analysis	Without magnesium sulfate With magnesium sulfate	Ref. 0.72 (0.58–0.89)	- 0.0028
Propensity-score models			
Adjusted for propensity score	Without magnesium sulfate With magnesium sulfate	Ref. 0.61 (0.49–0.76)	- <0.0001
With matching	Without magnesium sulfate With magnesium sulfate	Ref. 0.66 (0.51–0.84)	- 0.0007
With inverse probability weighting	Without magnesium sulfate With magnesium sulfate	Ref. 0.76 (0.60–0.95)	- 0.0153

Multivariable analysis adjusted for age, OASIS, AKI, sepsis, MV, CRRT, BUN, and creatinine.

Propensity score was calculated by age, OASIS, AKI, sepsis, MV, CRRT, BUN, and creatinine.

Propensity score matching was performed with the use of a 1:1 matching protocol without replacement (greedy-matching algorithm), with a caliper width equal to 0.05 of the standard deviation of the logit of the propensity score.

Inverse probability weighting was used with the same covariates according to the propensity score.

Abbreviations: OASIS, oxford acute severity of illness score; AKI, acute kidney injury; MV, mechanical ventilation; CRRT, continuous renal replacement therapy; BUN, blood urea nitrogen; HR, hazard ratio; CI, confidence interval.

### Stratified analyses and interaction tests

**FIGURE 3:**
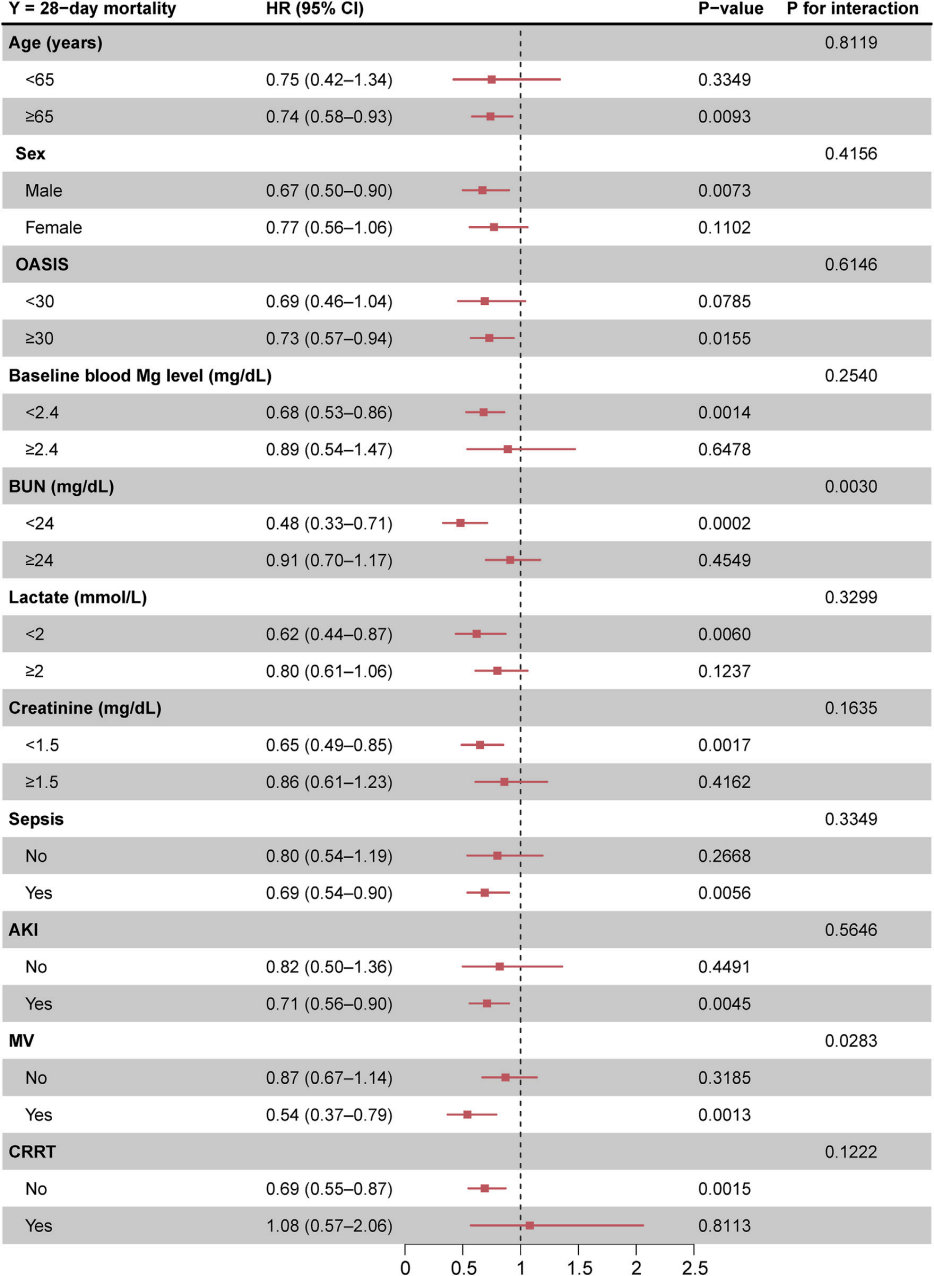
Forest plot of magnesium sulfate use on 28-day mortality in prespecified and exploratory subgroups in each subgroup. Abbreviations: HR, hazard ratio; CI, confidence interval; OASIS, Oxford acute severity of illness score; AKI, acute kidney injury; MV, mechanical ventilation; CRRT, continuous renal replacement therapy.

**FIGURE 4:**
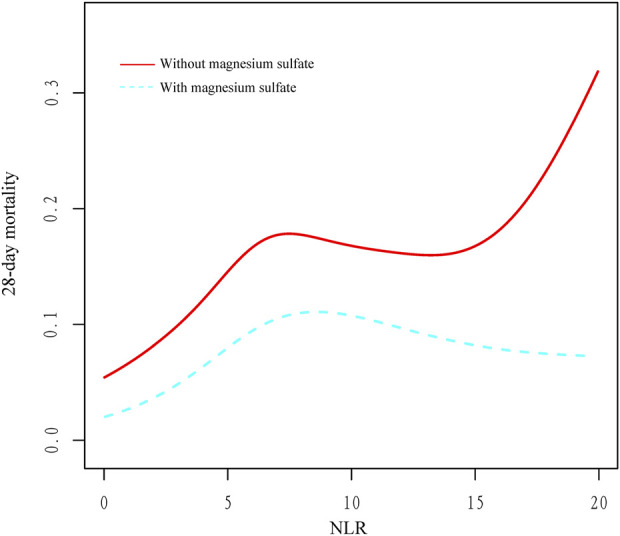
Relationship between NLR and 28-day mortality in critically ill patients with COPD. Abbreviations: NLR, neutrophil-to-lymphocyte ratio; COPD, chronic obstructive pulmonary disease.

The beneficial effects of magnesium sulfate on reducing 28-day mortality remained consistent across all subgroups analyzed Figure 3. Significant interactions were observed between magnesium sulfate administration and both BUN levels and mechanical ventilation use, with interaction p-values below 0.05, indicating statistical significance (Table 4). Specifically, patients with BUN levels under 24 mg/dL who received magnesium sulfate treatment exhibited a significantly reduced 28-day mortality rate (HR 0.48 [95% CI: 0.33–0.71]) compared to untreated patients. Similarly, a positive effect was noted in patients on mechanical ventilation who were treated with magnesium sulfate versus those who were not (HR = 0.54 [95% CI: 0.37–0.79]). These results highlight the substantial impact of magnesium sulfate in various clinical scenarios within ICU settings. Furthermore, while the relationship between magnesium sulfate use and 28-day mortality remained consistent across different baseline blood magnesium levels, no significant association was found in the subgroup with blood magnesium levels ≥2.4 mg/dL. Subgroup analyses of the neutrophil-to-lymphocyte ratio (NLR) and duration of mechanical ventilation indicated that patients treated with magnesium sulfate experienced lower 28-day mortality as NLR or duration of mechanical ventilation increased (Figure 4). Finally, we did not observe an increased risk of AKI associated with the early use of magnesium sulfate (Table 5).

**TABLE 4:** Effect size of magnesium sulfate use on 28-day mortality in prespecified and exploratory subgroups in each subgroup.

Y = 28-day mortality	Adjusted model			
	Without magnesium sulfate	With magnesium sulfate, HR (95% CI)	*P*-value	*P* for interaction
Age (years)				0.8119
<65	1.0	0.75 (0.42–1.34)	0.3349	
≥65	1.0	0.74 (0.58–0.93)	0.0093	
Sex				0.4156
Male	1.0	0.67 (0.50–0.90)	0.0073	
Female	1.0	0.77 (0.56–1.06)	0.1102	
OASIS				0.6146
<30	1.0	0.69 (0.46–1.04)	0.0785	
≥30	1.0	0.73 (0.57–0.94)	0.0155	
Baseline blood Mg level (mg/dL)				0.2540
<2.4	1.0	0.68 (0.53–0.86)	0.0014	
≥2.4	1.0	0.89 (0.54–1.47)	0.6478	
BUN (mg/dL)				0.0030
<24	1.0	0.48 (0.33–0.71)	0.0002	
≥24	1.0	0.91 (0.70–1.17)	0.4549	
Lactate (mmol/L)				0.3299
<2	1.0	0.62 (0.44–0.87)	0.0060	
≥2	1.0	0.80 (0.61–1.06)	0.1237	
Creatinine (mg/dL)				0.1635
<1.5	1.0	0.65 (0.49–0.85)	0.0017	
≥1.5	1.0	0.86 (0.61–1.23)	0.4162	
Sepsis				0.3349
No	1.0	0.80 (0.54–1.19)	0.2668	
Yes	1.0	0.69 (0.54–0.90)	0.0056	
AKI				0.5646
No	1.0	0.82 (0.50–1.36)	0.4491	
Yes	1.0	0.71 (0.56–0.90)	0.0045	
MV				0.0283
No	1.0	0.87 (0.67–1.14)	0.3185	
Yes	1.0	0.54 (0.37–0.79)	0.0013	
CRRT				0.1222
No	1.0	0.69 (0.55–0.87)	0.0015	
Yes	1.0	1.08 (0.57–2.06)	0.8113	

Note: Adjusted by age, OASIS, AKI, sepsis, MV, CRRT, BUN, and creatinine except for the subgroup variable.

Abbreviations: OASIS, oxford acute severity of illness score; AKI, acute kidney injury; MV, mechanical ventilation; CRRT, continuous renal replacement therapy; BUN, blood urea nitrogen.

**TABLE 5:** Association between magnesium sulfate use and AKI.

Organ failure			
AKI	Groups	OR (95% CI)	*P*-value
Crude model	Without magnesium sulfate With magnesium sulfate	Ref. 0.46 (0.40–0.54)	- <0.0001
Adjusted model	Without magnesium sulfate With magnesium sulfate	Ref. 0.55 (0.46–0.64)	- <0.0001

Note: Adjusted by age, sex, OASIS, charlson score, glucose, lactate; BUN, creatinine, and baseline blood Mg.

Abbreviations: OR, odds ratio; CI, confidence interval; OASIS, oxford acute severity of illness score; BUN, blood urea nitrogen; AKI, acute kidney injury.

## Discussion

In this study, we analyzed data from critically ill patients with COPD admitted to the ICU to investigate the relationship between magnesium sulfate administration within 48 h of ICU admission and clinical outcomes. The early administration of magnesium sulfate was associated with a lower 28-day mortality rate, without an increased risk of AKI. While the length of ICU stays showed no significant variance between the groups, patients receiving magnesium sulfate had a shorter duration of mechanical ventilation. These results imply that the benefits observed may be more closely tied to survival and respiratory improvement rather than disparities in overall resource utilization.

### Current clinical uses and research on magnesium sulfate

Magnesium sulfate is extensively utilized in conditions like preeclampsia, eclampsia, and specific arrhythmias. Its role in critical care has expanded recently, driven by emerging evidence of broader benefits. For example, magnesium has been investigated as a treatment for atrial fibrillation (to aid in restoring sinus rhythm), kidney protection, and neuroprotection in ICU patients (Gu et al., 2023; Davey and Teubner, 2005; Saver et al., 2015). A retrospective cohort study utilizing the MIMIC-IV database discovered that magnesium sulfate usage in patients with sepsis was linked to significantly lower 28-day mortality, a decreased incidence of AKI, and the necessity for renal replacement therapy (Roussos and Koutsoukou, 2003). These benefits are ascribed to magnesium’s anti-inflammatory and antioxidant properties, which might mitigate organ damage seen in critical illness. Importantly, these advantages seem to depend on maintaining magnesium within an optimal range. Another analysis observed that excessive magnesium supplementation resulting in hypermagnesemia (serum magnesium >1.07 mmol/L) was associated with increased 28- and 90-day mortality (Li et al., 2024). This highlights that while magnesium sulfate is generally well tolerated, oversupplementation (particularly in patients with renal impairment) can lead to adverse effects such as hypotension and respiratory depression. Therefore, precise dosing and monitoring of serum magnesium levels are crucial when using magnesium in critical care.

### Magnesium sulfate in COPD exacerbations: evidence and new insights

The effectiveness of magnesium sulfate in acute COPD exacerbation has been explored in various trials and reviews with varying outcomes (Jahangir et al., 2022; Shivanthan and Rajapakse, 2014; Skorodin et al., 1995). A recent Cochrane systematic review and other meta-analyses on intravenous magnesium in COPD indicated some modest advantages: magnesium therapy enhanced lung function (e.g., increased FEV_1_ and peak expiratory flow) and was linked to reduced odds of hospital admission (Ni et al., 2022). For instance, Jahangir et al. found that IV magnesium resulted in significantly improved FEV_1_/PEF outcomes and approximately a 55% decrease in the risk of hospitalization in COPD exacerbation patients compared to placebo (Jahangir et al., 2022). Magnesium has long been recognized for its beneficial effects on severe asthma attacks by alleviating bronchospasm and enhancing FEV_1_. However, it demonstrates limited efficacy in milder asthma exacerbations—a trend that appears to be somewhat mirrored in COPD research.

Despite these improvements in intermediate outcomes, previous randomized trials have not consistently shown improvements in the most critical endpoints of COPD exacerbation. Multiple RCTs, often small and single-center studies, have not demonstrated significant reductions in the need for ICU admission, intubation, or mortality with IV magnesium. For example, a trial involving 124 patients by Nouira et al. found no significant differences in intubation rates or 90-day mortality between the magnesium and control groups (Nouira et al., 2014). Similarly, an earlier trial by Skorodin et al. observed improved peak flow after magnesium administration but no significant impact on dyspnea scores or hospitalization rates (Skorodin et al., 1995). These inconsistent findings likely result from variations in study designs, such as differences in magnesium dosage (ranging from 1.2 g to 2.5 g), timing of administration, and patient populations, all of which can affect outcomes. Many RCTs have excluded the sickest COPD patients, such as those needing invasive mechanical ventilation or those with life-threatening respiratory failure. Therefore, previous trials may have lacked the power to detect survival benefits, particularly as they focused on moderate exacerbations treated in an emergency setting.

Our findings offer new insights in this context. In our analysis, encompassing a wide cohort of ICU patients with COPD exacerbation, the use of magnesium was linked to enhanced survival, particularly in the most severe cases necessitating mechanical ventilation. This differs from previous interventional trials that did not show a clear mortality benefit, underscoring that the life-saving potential of magnesium might only manifest in critical patient subgroups. In other words, magnesium sulfate could provide benefits in severe COPD exacerbations that were not evident in earlier studies focusing on less critical patients. By specifically investigating outcomes in mechanically ventilated individuals, our study indicates that magnesium might lower mortality rates where standard therapies often fall short. This new discovery warrants further exploration as it suggests that magnesium’s impact goes beyond minor improvements in lung function or hospital stay, potentially changing the trajectory of life-threatening COPD exacerbations.

### Potential mechanisms and benefits of magnesium sulfate

The physiological effects of magnesium sulfate offer plausible explanations for its potential benefits in critically ill patients with COPD. Magnesium is a well-known smooth muscle relaxant in the airways, acting by antagonizing calcium influx into bronchial smooth muscle cells and inhibiting acetylcholine release, resulting in bronchodilation (Barbagallo et al., 2021; Knightly et al., 2017). During severe exacerbations, magnesium administration can alleviate bronchospasm, reducing airway resistance and dynamic hyperinflation, thus enhancing ventilation efficiency. This effect likely contributes to improved gas exchange and may lower the risk of barotrauma or ventilator-induced lung injury by decreasing the pressure needed to ventilate stiff, constricted lungs. Maintaining adequate magnesium levels has been linked to a shorter duration of mechanical ventilation in ICU patients, suggesting that magnesium-facilitated bronchodilation and lung mechanics can expedite respiratory recovery (Altun et al., 2019; Santosh Raju et al., 2023). Additionally, magnesium exhibits central nervous system and neuromuscular effects that could enhance patient-ventilator synchrony. By blocking N-methyl-D-aspartic acid receptors and reducing acetylcholine release at the neuromuscular junction, magnesium imparts mild sedative and muscle relaxant properties (Wang et al., 2011; Laires et al., 2004). These effects may help prevent episodes of dyssynchrony in mechanically ventilated patients. By promoting a calmer respiratory drive and relaxing respiratory muscles, magnesium might reduce the need for deep sedation or paralytics to achieve ventilator synchrony. Supporting this, a clinical study demonstrated that adding magnesium infusion to standard sedatives in critically ill patients significantly shortened the duration of mechanical ventilation and decreased sedative requirements (Altun et al., 2019). Improved ventilator synchrony and earlier weaning can lead to fewer ventilator-associated complications (such as ventilator-induced lung injury, nosocomial pneumonia, and prolonged sedation delirium) and ultimately enhance survival. Furthermore, the anti-inflammatory and antioxidant properties of magnesium are particularly relevant in COPD exacerbations, which are often characterized by intense airway and systemic inflammation. Magnesium can inhibit the release of pro-inflammatory cytokines such as TNF-α and IL-6, thereby attenuating the inflammatory cascade (Aryana et al., 2014; Cross et al., 2018). In patients with sepsis, this immunomodulation has been associated with improved organ function and reduced mortality; similar benefits may be observed in COPD exacerbations, where inflammation contributes to respiratory failure (Jain et al., 2024). Magnesium also acts as a cofactor for enzymes involved in scavenging reactive oxygen species, thereby reducing oxidative stress and tissue damage in the lungs (Liu and Dudley, 2020). By mitigating inflammation and oxidative injury, magnesium can help protect against multiorgan dysfunction during severe exacerbations and enhance the overall resilience of the patient.

In summary, the multifaceted actions of magnesium sulfate, including bronchodilation, modulation of the respiratory drive, and anti-inflammatory and antioxidant effects, provide a strong theoretical basis for the observed mortality reduction in patients with mechanically ventilated COPD. It is likely to improve acute respiratory physiology (facilitating ventilation and oxygenation) while addressing the systemic factors (inflammation and oxidative stress) that influence recovery. Careful monitoring is imperative, and even as we harness the therapeutic effects of magnesium, we must avoid hypermagnesemia (>2.4 mg/dL, or >1.0 mmol/L), which can cause hypotension, bradyarrhythmia, and respiratory depression. When used judiciously, magnesium sulfate has emerged as a promising adjunct in the management of severe COPD exacerbations, especially in patients requiring invasive ventilation, by improving both the pulmonary and systemic conditions that determine their outcomes. This hypothesis, supported by our data, adds a new dimension to the discussion on magnesium in COPD care and encourages future prospective trials targeting this high-risk population.

### Study limitations and future directions

This study has several limitations. First, its retrospective single-center design inherently carries the risk of selection bias, limiting its generalizability to other institutions or healthcare systems. Despite applying multiple propensity-score-based methods and calculating E-values, our results remained susceptible to residual confounding from unmeasured variables. Specifically, the E-value analysis indicated that an unmeasured confounder would require a risk ratio of at least 1.96 with both treatment allocation and outcome to fully explain the observed association, suggesting that a substantial hidden bias would be necessary to nullify our findings. Second, detailed data on the exact timing of magnesium sulfate administration relative to key interventions, such as the initiation of mechanical ventilation, were not systematically recorded, precluding a more granular temporal analysis. Additionally, the administered dose of magnesium sulfate was not standardized; most patients received approximately 2 g intravenously; however, variations in dosing and duration prevented a meaningful dose–response evaluation. Third, we only extracted baseline serum magnesium levels measured within the first 24 h of ICU admission without information on intratherapy or post-therapy levels. Consequently, we could not assess dynamic changes in serum magnesium levels or the incidence of hypermagnesemia over the course of treatment. Fourth, potentially relevant concomitant medications such as corticosteroids, bronchodilators, antibiotics, and diuretics were not consistently available in the database and were thus not included in our propensity score models. Furthermore, certain disease severity indicators beyond the OASIS score, such as PaO_2_/FiO_2_ ratios or detailed ventilator parameters, were either incomplete or unavailable. Although our machine learning-based covariate selection and propensity-based adjustments were designed to mitigate such imbalances, the absence of these variables may introduce bias. Finally, the observational nature of the study precludes causal inferences. The findings should be interpreted as hypothesis-generating rather than definitive evidence of benefits.

Future research should aim to validate these results in multicenter prospective trials that incorporate standardized dosing protocols, serial magnesium concentration monitoring, and detailed temporal mapping of administration relative to critical interventions. Such studies should also explore subgroup effects, particularly in mechanically ventilated patients and those with varying baseline magnesium levels or renal functions, to refine patient selection and optimize the efficacy and safety profile of magnesium sulfate for ICU-managed COPD exacerbations.

## Conclusion

In this single-center retrospective cohort study, the early administration of magnesium sulfate in critically ill patients with COPD was associated with lower 28-day mortality. This association remained consistent across baseline serum magnesium levels and did not increase the risk of AKI. These findings imply a potential therapeutic role for magnesium sulfate in this population, particularly in severe cases necessitating intensive care. However, causal inferences cannot be drawn due to the observational design. Further multicenter prospective studies with standardized dosing, serial magnesium monitoring, and comprehensive subgroup analyses are needed to validate these associations and establish evidence-based guidelines for the optimal use of magnesium sulfate in critically ill patients with COPD.

## Data Availability

The raw data supporting the conclusions of this article are from a third-party dataset available from MIMIC-IV, a freely accessible critical care database. Reproduction of these data is not permitted according to the Data Use Agreement of the database, but access can be requested here: https://mimic.physionet.org/gettingstarted/access.
